# Melatonin and *Leishmania amazonensis* Infection Altered miR-294, miR-30e, and miR-302d Impacting on *Tnf*, *Mcp-1*, and *Nos*2 Expression

**DOI:** 10.3389/fcimb.2019.00060

**Published:** 2019-03-20

**Authors:** Juliane Cristina Ribeiro Fernandes, Juliana Ide Aoki, Stephanie Maia Acuña, Ricardo Andrade Zampieri, Regina P. Markus, Lucile Maria Floeter-Winter, Sandra Marcia Muxel

**Affiliations:** ^1^Departamento de Fisiologia, Instituto de Biociências, Universidade de São Paulo, São Paulo, Brazil; ^2^Instituto de Medicina Tropical, Universidade de São Paulo, São Paulo, Brazil

**Keywords:** polyamine pathway, nitric oxide synthase, arginase 1, interleukin, mRNA-miRNA interaction, melatonin and *Leishmania*

## Abstract

Leishmaniases are neglected diseases that cause a large spectrum of clinical manifestations, from cutaneous to visceral lesions. The initial steps of the inflammatory response involve the phagocytosis of *Leishmania* and the parasite replication inside the macrophage phagolysosome. Melatonin, the darkness-signaling hormone, is involved in modulation of macrophage activation during infectious diseases, controlling the inflammatory response against parasites. In this work, we showed that exogenous melatonin treatment of BALB/c macrophages reduced *Leishmania amazonensis* infection and modulated host microRNA (miRNA) expression profile, as well as cytokine production such as IL-6, MCP-1/CCL2, and, RANTES/CCL9. The role of one of the regulated miRNA (miR-294-3p) in *L. amazonensis* BALB/c infection was confirmed with miRNA inhibition assays, which led to increased expression levels of *Tnf* and *Mcp-*1/*Ccl2* and diminished infectivity. Additionally, melatonin treatment or miR-30e-5p and miR-302d-3p inhibition increased nitric oxide synthase 2 (*Nos2*) mRNA expression levels and nitric oxide (NO) production, altering the macrophage activation state and reducing infection. Altogether, these data demonstrated the impact of melatonin treatment on the miRNA profile of BALB/c macrophage infected with *L. amazonensis* defining the infection outcome.

## Introduction

Leishmaniases are neglected tropical diseases characterized by cutaneous, mucocutaneous, or visceral lesions (Alvar et al., 2012; Scott and Novais, 2016). The diseases are endemic in 98 countries worldwide (Alvar et al., 2012). According to the World Health Organization (WHO), approximately 12 million people are currently infected, and ~20,000–30,000 deaths occur annually (Alvar et al., 2012; WHO, 2017). The etiological agents of leishmaniases are the protozoan parasites of *Leishmania* genus (Marsden, 1986; Ashford, 2000).

*Leishmania* amastigotes are obligatory intracellular parasites, which survive and replicate inside macrophage phagolysosomes, being able to modulate the host immune response by the reduction of inflammation and the development of an adaptive immune response (Nathan and Shiloh, 2000; Gregory and Olivier, 2005; Mosser and Edwards, 2008; Scott and Novais, 2016). Modulation of type 1 (Th1) or type 2 (Th2) polarization of T CD4^+^ lymphocytes is essential to define the fate of infection, inducing death or proliferation of *Leishmania* amastigotes in the macrophages (Corraliza et al., 1995; Munder et al., 1998; Wanasen and Soong, 2008). Some enzymes, such as nitric oxide synthase 2 (NOS2) and arginase 1 (ARG1), are competitively regulated by Th1 or Th2 cytokines, and both enzymes use L-arginine as substrate. Stimulation with Th1-associated cytokines and chemokines, such as interferon gamma (IFN-γ), tumor necrosis factor (TNF) and granulocyte macrophage colony-stimulating factor (GM-CSF), polarize macrophages to the M1 phenotype by increasing NOS2 and decreasing ARG1 levels, leading to parasite control (Hrabak et al., 1996; Boucher et al., 1999; Mantovani et al., 2004; Wang et al., 2014). Conversely, Th2-associated cytokines and chemokines, such as interleukin 4 (IL-4), IL-13, tumor growth factor beta (TGF-β), IL-10 and macrophage colony-stimulating factor (M-CSF) (Verreck et al., 2004), induce M2 polarization by decreasing NOS2 and increasing ARG1 levels (Martinez et al., 2009), leading to parasite replication and survival (Hrabak et al., 1996; Boucher et al., 1999; Mantovani et al., 2004; Wang et al., 2014).

Melatonin, the darkness hormone, is synthesized during the night by the pineal gland under the control of the central clock, the suprachiasmatic nuclei of the hypothalamus. Melatonin is also synthesized by immune-competent cells and plays a role in surveillance against infection and in the recovery phase of acute defense responses (Markus et al., 2007, 2017; Carrillo-Vico et al., 2013). The interplay between timing and defense is a fine-tuned regulated process that involves melatonin-mediated restriction of leukocyte migration from the circulation to the tissues under normal conditions (Lotufo et al., 2001; Ren et al., 2015). However, suppression of pineal melatonin synthesis can occur in response to pathogen (bacteria and fungi)- and danger-associated molecular patterns (Da Silveira Cruz-Machado et al., 2010; Carvalho-Sousa et al., 2011) to allow migration of leukocytes to the lesion site at night as well as during the day. In addition, melatonin can be synthesized “on demand” by macrophages (Pontes et al., 2006; Muxel et al., 2012), dendritic cells (Pires-Lapa et al., 2018) and lymphocytes (Carrillo-Vico et al., 2004) during the recovery phase or under low-grade and chronic inflammatory conditions (Markus et al., 2017). This fine-tuned regulation of melatonin synthesis reduces susceptibility to bacterial infection (Rojas et al., 2002), lethal endotoxemia (Maestroni, 1996; Prendergast et al., 2003) and several parasite infections such as *Schistosoma mansoni* (El-Sokkary et al., 2002), *Plasmodium falciparum*, and *Plasmodium chabaudi* (Hotta et al., 2000), *Trypanosoma cruzi* (Santello et al., 2007), *Leishmania infantum* (Elmahallawy et al., 2014), and *Leishmania amazonensis* (Laranjeira-Silva et al., 2015). Melatonin promotes the expression of the immunoregulatory phenotype in immune-competent cells (Rojas et al., 2002; Kinsey et al., 2003), acting as a cytoprotector (Luchetti et al., 2010), an antioxidant (Reiter et al., 2013; Zhang and Zhang, 2014) and an immunomodulator (Reiter et al., 2000; Carrillo-Vico et al., 2005, 2013). Unlike bacteria and fungi, *L. amazonensis* did not suppress the nocturnal melatonin surge (Laranjeira-Silva et al., 2015), resulting in lower infectivity in the dark environment than in the day.

The immune response can also be modulated by microRNAs (miRNAs) participation (Baltimore et al., 2008; O'neill et al., 2011; Muxel et al., 2017b, 2018b). miRNAs are small non-coding RNAs that act as posttranscriptional regulators by targeting messenger RNAs (mRNAs) via 3′ untranslated region (UTR) sequence complementarity, leading to translational repression or mRNA degradation, among other mechanisms (Bagga et al., 2005; Lim et al., 2005). miRNAs are involved in macrophage activation and polarization (Baltimore et al., 2008; Graff et al., 2012; Banerjee et al., 2013; Wang et al., 2014). In recent years, some studies have been describing macrophage miRNA modulation during *Leishmania* infections (Ghosh et al., 2013; Lemaire et al., 2013; Frank et al., 2015; Geraci et al., 2015; Mukherjee et al., 2015; Muxel et al., 2017b). In this study, we demonstrated that the miRNA profile of *Leishmania*-infected macrophages was modified after melatonin treatment. Also, melatonin reduced the levels of cytokines and chemokines such as IL-6, MCP-1, MIP-2/CXCL2, and RANTES/CCL5. In contrast, melatonin increased *Nos2* mRNA expression and NO production during infection. Melatonin treatment, as well as functional inhibition of miRNAs in macrophages, impaired the infectivity of *L. amazonensis*.

## Materials and Methods

### Parasite Culture

*Leishmania amazonensis* (MHOM/BR/1973/M2269) promastigotes were maintained in culture at 25°C in M199 medium (Invitrogen, Grand Island, NY, USA), supplemented with 10% heat-inactivated fetal bovine serum (FBS, Invitrogen), 5 ppm hemine, 100 μM adenine, 10 U/mL penicillin (Invitrogen), 10 μg/mL streptomycin (Life Technologies, Carlsbad, CA, USA), 40 mM HEPES-NaOH and 12 mM NaHCO_3_ buffer (pH 6.85). The cultures were maintained for 7 days until the new subcultures and only in early passages (P1–P5) for infection assays.

### *In vitro* Macrophage Infection

All experiments were performed with 6–8 weeks-old female BALB/c mice obtained from the Animal Center of the Institute of Bioscience of the University of São Paulo. Bone marrow-derived macrophages (BMDMs) were obtained from femurs and tibias by flushing with 2 mL of PBS. Then, the collected cells were centrifuged at 500 × g for 10 min at 4°C and resuspended in RPMI 1640 medium (LGC Biotecnologia, São Paulo, Brazil) supplemented with penicillin (100 U/ml) (Invitrogen, São Paulo, Brazil), streptomycin (100 μg/ml) (Life Technologies, Carlsbad, CA, USA), 10% heat-inactivated FBS (Invitrogen) and 20% of L929 cell supernatant. The cells were submitted to differentiation for 7–8 days at 34°C in an atmosphere of 5% CO_2_. BMDMs were used after phenotypic analysis by flow cytometry showing at least 95% F4/80- and CD11b-positive cells. Fluorescence detection was performed using an Amnis FlowSight (Merck-Millipore, Darmstadt, Germany) and analyzed using Ideas® Software (Amnis Corporation, Seattle, WA, USA).

For melatonin treatment assays, 2 × 10^5^ cells were plated into 8-wells glass chamber slides (Lab-Teck Chamber Slide; Nunc, Naperville, IL, USA) and incubated at 34°C in an atmosphere of 5% CO_2._ After, macrophages were treated with 3 or 30 nM melatonin (Tocris, Bristol, United Kingdom), vehicle (0.0005% ethanol in medium, Sigma-Aldrich, St. Louis, MO, USA) or medium only (untreated control) for 1, 2, or 4 h. Then, the macrophages were infected with promastigotes in the stationary growth phase (MOI 5:1), as previously described (Laranjeira-Silva et al., 2015). After 4 h of infection, the cells were washed to remove nonphagocytosed promastigotes, and then incubated with fresh RPMI medium supplemented as previously described, or removed for cell-fixation process. The infectivity was microscopically analyzed after 4 and 24 h of infection, cell-fixation was performed with acetone/methanol (1:1, v:v, Merck, Darmstadt, Germany) for 20 min at −20°C, followed by PBS washing and Panoptic-staining (Laborclin, Parana, Brazil). Infectivity was analyzed in phase-contrast microscopy (Nikon Eclipse E200, NJ, USA) counting the number of infected macrophages and amastigotes per macrophage in at least 1,000 macrophages/treatment in three independent experiments. The infection index was calculated by multiplying the mean number of amastigotes per macrophage by the rate of macrophage infection. The values were normalized based on the average values for the untreated infected macrophages.

For mRNA and miRNA expression analysis, 5 × 10^6^ cells/well were plated into 6-well plates (SPL Life Sciences, Pocheon, Korea) and for cytokines quantification in supernatant, 1 × 10^6^ cells/well were plated into 24-well plates (SPL Life Sciences), then the melatonin treatment (30 nM for 4 h before infection) and infection were performed as described above.

### RNA Extraction, Reverse Transcription, and RT-qPCR for miRNA

Total RNA was extracted using a miRNeasy Mini Kit (Qiagen, Hilden, Germany) following the manufacturer's instructions. cDNA was synthesized from mature miRNA templates using a miScript II RT Kit (Qiagen), according the manufacturer's instructions. Briefly, 250 ng of total RNA was added to 2 μL of 5X miScript HiSpec Buffer, 1 μL of 10X Nucleics Mix and 1 μL of miScript Reverse Transcriptase Mix. RNase-free water was added to a final volume of 10 μL. The RNA was incubated for 60 min at 37°C to insert poly-A tail downstream of the miRNA sequence and to anneal a T-tail tag for the elongation of the cDNA. The enzyme was inactivated at 95°C for 5 min. The reaction was performed in Mastercycler Gradient thermocycler (Eppendorf, Hamburg, Germany), and the product was stored at −20°C until use.

An array of 84 miRNAs was measured using a Mouse Inflammatory Response and Autoimmunity miRNA PCR Array kit (MIMM-105Z, Qiagen) and miScript SYBR PCR Kit (Qiagen). The reaction was performed with 2X QuantiTect SYBR Green PCR Master Mix, 10X miScript Universal Primer and 105 μL of cDNA (triplicate samples of the 10-fold diluted cDNA). RNase-free water was added to a final volume of 2,625 μL (25 μL/well).

For specific amplification of miR-181c, miR-294-3p, miR-30e, miR-302d, and SNORD95A (used as a normalizer), reactions were prepared with 2X QuantiTect SYBR Green PCR Master Mix, 10X miScript Universal Primer, 10X specific primer, 5 μL of cDNA (3–4 samples 10-fold diluted) and RNase-free water to a final volume of 25 μL/well. qPCR started with activation of the HotStart DNA Polymerase for 15 s at 95°C and 40 cycles of 15 s at 94°C for denaturation, 30 s at 55°C for primer annealing and 30 s at 70°C for elongation. The reaction was performed in Thermocycler ABI Prism 7300 (Applied Biosystems, Carlsbad, CA, USA), and the relative Ct was analyzed using online tools provided with the kit (miScript miRNA PCR Array Data Analysis software). The geometric average Ct of the miRNAs was normalized based on the SNORD95A values, and then the fold changes were calculated to compare untreated, vehicle-treated or melatonin-treated and infected macrophages in relation to untreated and uninfected macrophages at the same time of culture. The RT-qPCR efficiencies were determined and a negative control reaction without reverse transcriptase enzyme was included to verify the absence of any DNA contamination in the RNA samples. The fold regulation (FR) was considered to be the negative inverse of the fold change [function = −1^*^(1/fold change value)]. FR ≥ 1.5 were considered to indicate upregulation, and levels ≤ −1.5 were considered to indicate downregulation, as previously described (Muxel et al., 2017a).

### Reverse Transcription and RT-qPCR for mRNA

Reverse transcription was performed using 2 μg of RNA and 20 nmol of random primer (Applied Biosystems) to a final volume of 13 μL. The mixture was incubated at 70°C for 5 min and then at 15°C for addition of the mix including 4 μL of 5X buffer, 2 μL of 10 mM dNTPs and 1 μL (2U) of RevertAid™ Reverse Transcriptase (Fermentas Life Sciences, Burlington, Ontario, Canada). The reaction was incubated at 37°C for 5 min and at 42°C for 60 min. The enzyme activity was blocked by heat inactivation at 75°C for 15 min, and the cDNA was stored at −20°C until use. A negative control reaction without reverse transcriptase enzyme was included to verify the presence of some DNA contamination in the RNA samples. Reactions were performed with 2X SYBR Green PCR Master Mix (Applied Biosystems), 200 nM of each primer pair, 5 μL of cDNA (100-fold diluted) and RNase-free water to a final volume of 25 μL. The reactions were performed in an Exicycler™ 96 Real-Time Quantitative Thermal Block (Bioneer, Daejeon, Korea). The mixture was incubated at 94°C for 5 min followed by 40 cycles of 94°C for 30 s and 60°C for 30 s. Quantification of target gene expression was performed based on a standard curve prepared from a 10-fold serial dilution of a quantified and linearized plasmid containing the target DNA. The following primer pairs were used for mouse mRNA analysis: *Nos2*: 5′-agagccacagtcctctttgc-3′ and 5′-gctcctcttccaaggtgctt-3′; *Arg1:* 5-agcactgaggaaagctggtc-3′ and 5′-cagaccgtgggttcttcaca-3′; *Cat-2b:* 5′-tatgttgtctcggcaggctc-3′ and 5′-gaaaagcaacccatcctccg-3′; *Cat1:* 5′-cgtaatcgccactgtgacct-3′ and 5′-ggctggtaccgtaagaccaa-3′; *Mcp-1/Ccl2:* 5′-tgatcccaatgagtaggctgg-3′ and 5′-gcacagacctctctcttgagc-3′, *Rantes/Ccl5:* 5′- ggagtatttctacaccagcagca-3′ and 5′-cccacttcttctctgggttgg-3′, *Tnf:* 5′- ccaccacgctcttctgtcta−3′ and 5′-agggtctgggccatagaact−3′ and *Gapdh*: 5′-ggcaaattcaacggcacagt-3′ and 5′-ccttttggctccacccttca-3′.

### Transfection of miRNA Inhibitors

For miRNA expression analysis, 5 × 10^5^ cells/well were plated into 24-well plates (SPL Life Sciences, Pocheon, Korea) and incubated at 34°C in an atmosphere of 5% CO_2_. For infectivity analysis, 2 × 10^5^ cells/well were plated into 8-well glass Lab-Teck chamber slides (Thermo Scientific, NY, USA) and incubated at 34°C in an atmosphere of 5% CO_2_ for 18 h. Then, the cells were incubated with 30 or 100 nM of the inhibitors miR-181c-5p, miR-294-3p, miR-30e-5p, miR-302d-3p, the negative control (Ambion, Carlsbad, CA, USA) or only medium (untreated), which were previously incubated for 20 min at room temperature with 3 μL of the FuGENE HD transfection reagent (Roche, Madison, WI, USA) in 250 μL of 10% FBS RPMI 1640 medium (LGC Biotecnologia, São Paulo, Brazil). After 36 h of transfection, the cells were infected, as previously described.

### Cytokine Quantification

The cytokines IL-1α, IL-1β, IL-4, IL-6, TNF-α, IL-13, IL-10, and IL-12; and the chemokines RANTES/CCL5, KC/CXCL1, MIP-2/CXCL2 and MCP-1/CCL2 were quantified using 25 μL of the supernatant of 1 × 10^6^ cells of uninfected or infected macrophages that were untreated, vehicle-treated or melatonin-treated using a MILLIPLEX MAP Mouse Cytokine/Chemokine Panel I kit (Merck Millipore, MA, USA), according to the manufacturer's instructions.

### NO Quantification

NO quantification was performed with DAF-FM (4-amino-5-methylamino-2′,7′-difluorofluorescein diacetate; Life Technologies, Eugene, OR, USA) labeling and analyzed by flow cytometry (FlowSight, Merck Millipore, Germany), as previously described (Muxel et al., 2017a,b).

### *In silico* Analysis

To analyze miRNA-mRNA interactions, we used the miRecords platform (http://c1.accurascience.com/miRecords/), which provides information of predicted mRNA targets by integrating data from various tools: DIANA-microT, MicroInspector, miRanda, MirTarget2, miTarget, NBmiRTar, PicTar, PITA, RNA22, RNAhybrid, and TargetScan/TargetScanS.

### Statistical Analysis

Statistical analyses were performed using GraphPad Prism Software (GraphPad Software Inc, La Jolla, CA, USA). Significance was determined based on Student's *t*-test and *p* < 0.05 was considered significant.

## Results

### Melatonin Reduces macrophage- *Leishmania amazonensis* Infectivity in a Dose- and time-Dependent Manner

**Figure 1:**
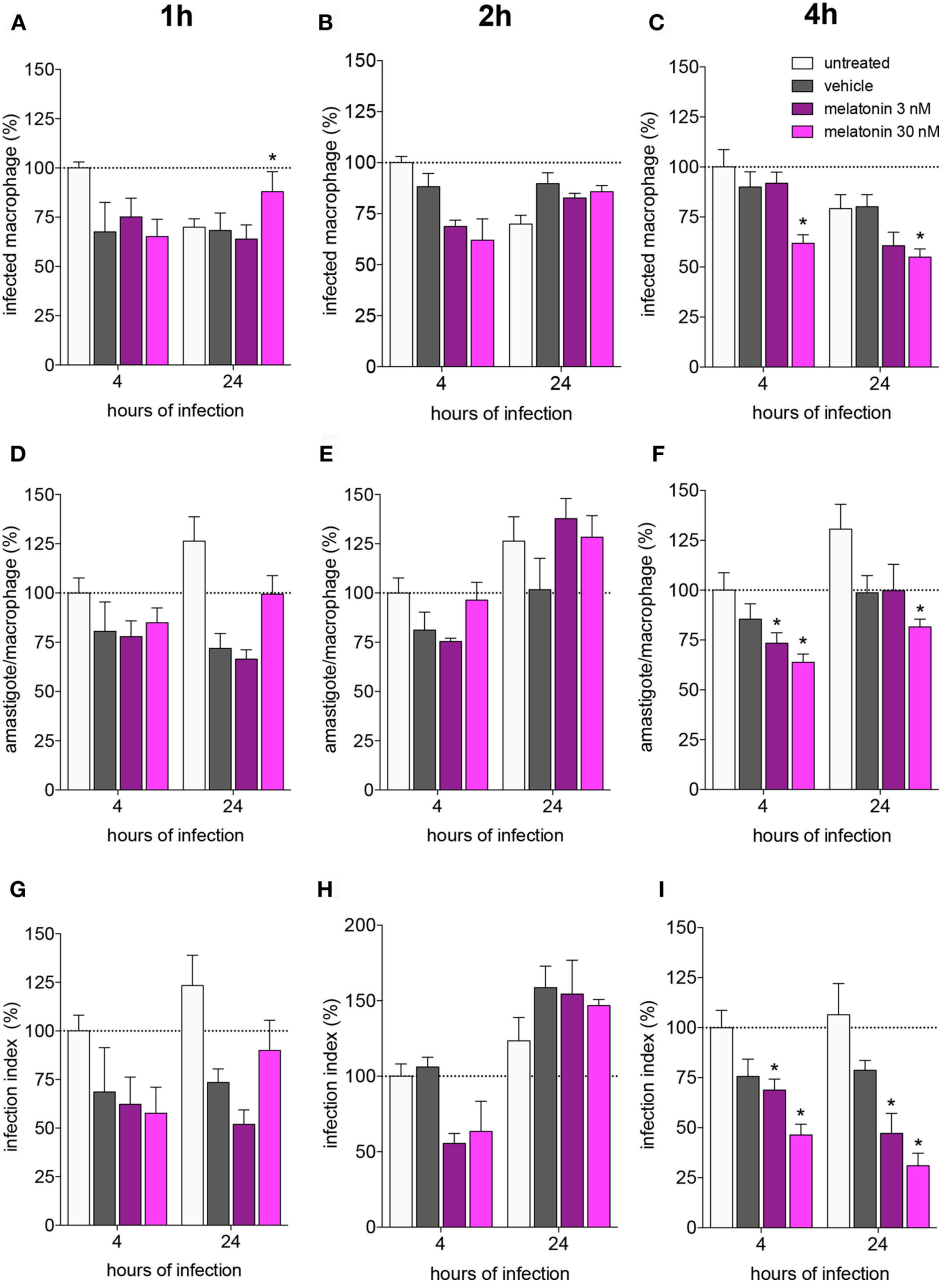
Melatonin inhibition of *L. amazonensis* infectivity in a dose dependent manner. BALB/c macrophages (2 × 10^5^ cells) were pre-incubated for 1, 2, or 4 h with medium (untreated, white bar), vehicle (ethanol 0.0005%, gray bar), or 3 (purple bar) or 30 (magenta bar) nM of melatonin. After, macrophages were infected with *L. amazonensis* (MOI 5:1) and analyzed after 4 and 24 h. **(A–C)**—Percentage of infected macrophages; **(D–F)**—number of amastigotes per infected macrophage; **(G–I)**—Infection index (rate of infected macrophages multiplied by the number of amastigotes per infected macrophage). Each bar represents the mean ± SEM of values normalized by untreated macrophages at 4 h of infection. The data were representative of three independent experiments (*n* = 5–8). ^*^p < 0.05, comparing the melatonin treatment with the vehicle-treated macrophage at the same concentration and time.

Melatonin treatment (3 or 30 nM) for 1 and 2 h did not modify the macrophage infection rate (as showed in the normalized data in Figures 1A,B and original data in Supplementary Figure 1), mean number of amastigotes per infected macrophage (3–5 amastigotes. Figures 1D,E) or infection index (Figures 1G,H) after 4 and 24 h of infection, compared to vehicle treatment.

Otherwise, melatonin (30 nM) treatment for 4 h showed reduction of 30% in the number of infected macrophages, while treatment with 3 nM of melatonin had no effect (Figure 1C; Supplementary Figure 1). The number of amastigotes per infected cell was reduced by the same percentage (20–25%) with 3 or 30 nM (Figure 1F), and the infection index was reduced at both concentrations (20–30% 3 nM, 60% 30 nM; Figure 1I). Subsequent analyses were performed based on treatment with 30 nM melatonin for 4 h.

### Melatonin Modulates the Expression of Genes Related to L-arginine Transport and Metabolism in *Leishmania amazonensis*-Infected Macrophages

**Figure 2:**
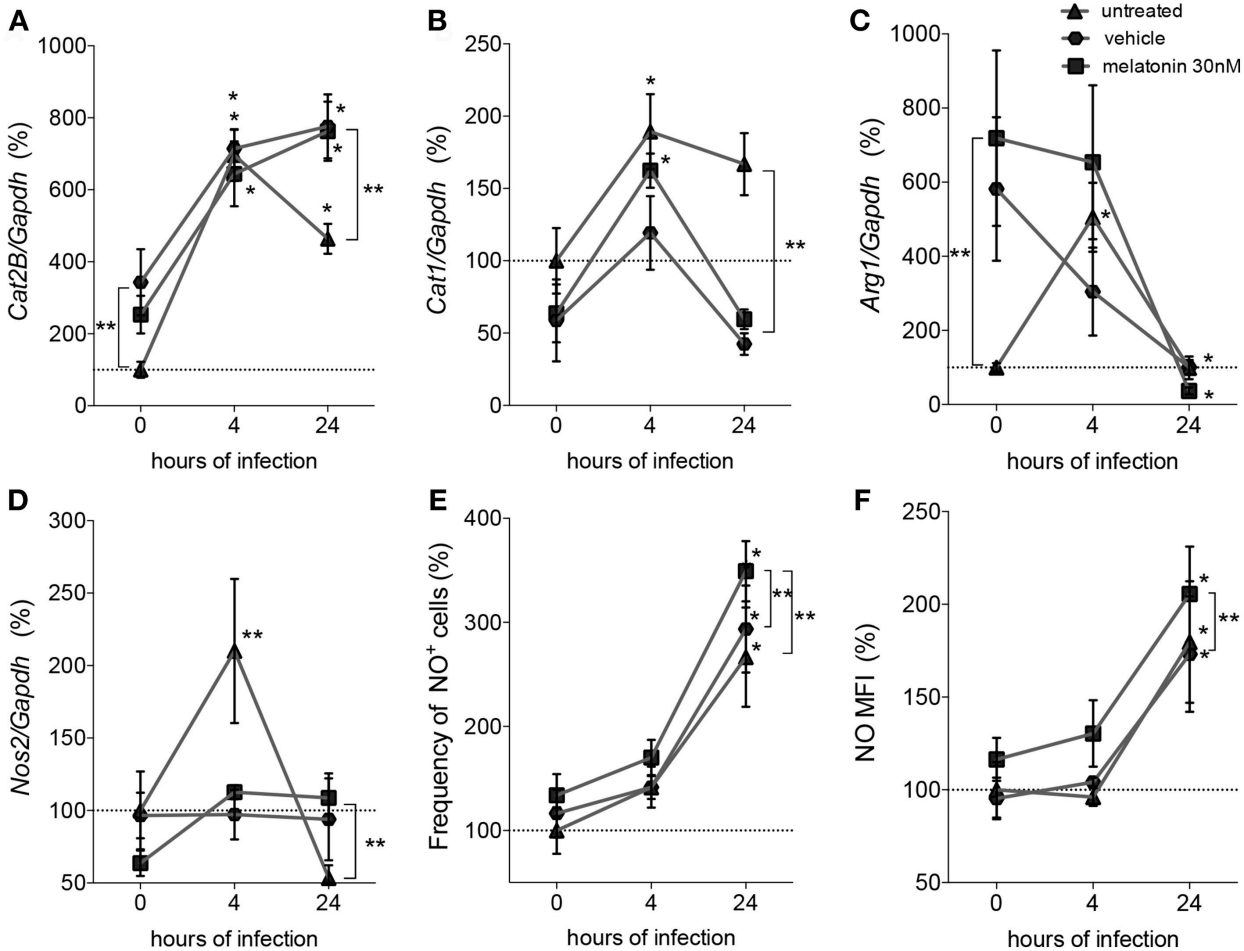
Quantification of mRNA involved in L-arginine transport and metabolism and NO production. Macrophages (5 × 10^6^) were treated with 30 nM of melatonin (squared dots), vehicle (5 × 10^−6^% ethanol, hexagonal dots) or untreated (triangular dots) for 4 h, infected with *L. amazonensis* (MOI 5:1) and collected after 4 and 24 h of infection. RT-qPCR of *Cat2B* **(A)**, *Cat1* **(B)**, *Arg1* **(C)**, and *Nos2* **(D)** were normalized by *Gapdh* quantification and the values were normalized by untreated-uninfected condition (0 h); **(E)** Frequency of NO producing cells and **(F)** mean of fluorescence intensity (MFI) of NO were quantified by DAF-FM label using flow cytometry. Each bar represents the average ± SEM of the values obtained in three independent experiments (*n* = 6–8). Statistical significance was determined based on two-tailed Student's *t*-test. ^*^p < 0.05, infected macrophages (4 h or 24 h) compared to non-infected (0 h) ^**^p < 0.05, melatonin-treated compared to untreated or vehicle.

Considering that parasite survival in macrophages is affected by deviation of L-arginine metabolism to the production of polyamines (Muxel et al., 2018a), we evaluated the gene expression of melatonin-treated macrophages in comparison with vehicle-treated macrophages, by quantification of mRNA expression levels of *Cat2B* and *Cat1* (both involved in the macrophage L-arginine uptake), and of *Arg1* and *Nos2* (involved in the polyamine production and NO production, respectively) (Figure 2).

Based on these mRNA expression levels, we observed that at both times of infection, early (4 h) and established (24 h), *L. amazonensis* induced sustained expression of *Cat2B* and *Cat1* mRNA (Figures 2A,B). *Arg1* and *Nos*2 were transiently expressed in early (4 h) infected macrophages compared to uninfected macrophages. Exposure to vehicle and melatonin modulated mRNA levels in uninfected macrophages, increasing the basal levels of *Cat2B* and *Arg1* (Figures 2A–C). However, in infected macrophages, melatonin treatment did not alter the *Cat2B, Cat1*, or *Arg*1 mRNA levels (Figures 2A–C) compared to vehicle treatment. Still, melatonin sustained the levels of *Nos2* mRNA (Figure 2D) and increased the frequency of NO-producing cells (Figure 2E) and the amount of NO per cell (Figure 2F) at 24 h of infection. Our data revealed that melatonin treatment of macrophages promoted modulation of the expression of mRNA involved in L-arginine metabolism during infection, inducing *Nos2* to the detriment of *Arg1* and thus altering infectivity.

### Melatonin Modifies Cytokine and Chemokine Production in *Leishmania amazonensis*-Infected Macrophages

**Figure 3:**
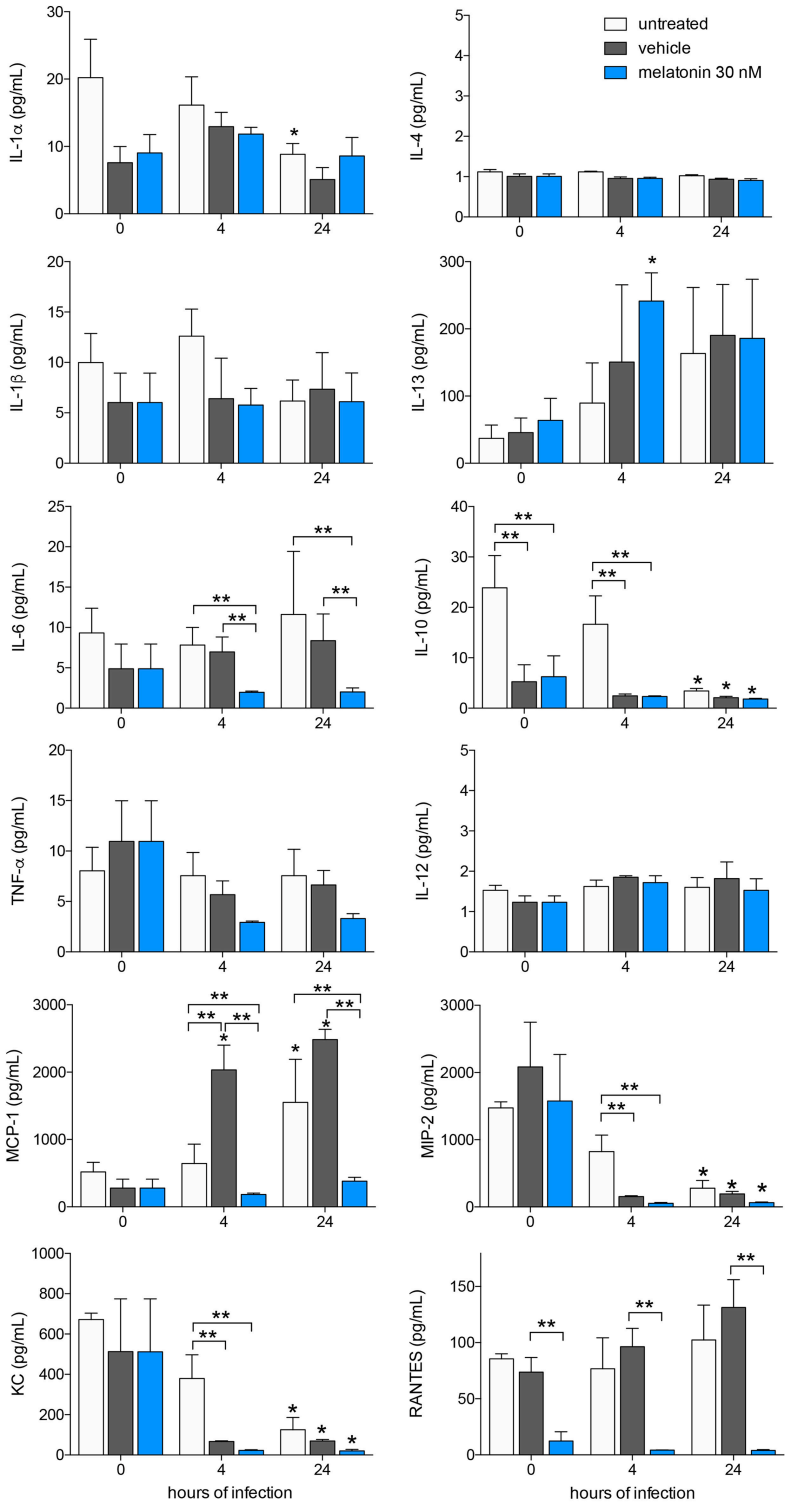
Cytokine and chemokines production after melatonin treatment and *L. amazonensis* infection. Cytokine and chemokine quantifications were performed with macrophage supernatants (1 × 10^6^/500 μL) treated with melatonin (30 nM, blue bar), vehicle (5 × 10^−6^% ethanol, gray bar) or untreated (white bar) for 4 h, infected with *L. amazonensis* (MOI 5:1) and collected after 4 and 24 h of infection. The time 0 corresponds to non-infected macrophages. Each bar represents medium ± SEM of values obtained in three independent experiments (*n* = 4–5). ^*^p < 0.05, infected compared to uninfected macrophages (0 h) ^**^p < 0.05, melatonin-treated compared to untreated or vehicle.

Furthermore, we analyzed cytokine and chemokine production in response to melatonin treatment and *L. amazonensis* infection. The levels of the cytokines IL-4, IL-1β, IL-13, IL-6, TNF-α, and IL-12 and the chemokine RANTES/CCL5 did not changed after 4 and 24 h of infection, whereas IL-1α (2-fold), IL-10 (5-fold), MIP-2/CXCL2 (8-fold), and KC/CXCL1 (4-fold) showed decreased levels after 24 h of infection compared to those in uninfected macrophages (Figure 3). In contrast, MCP-1/CCL2 (2-fold) showed increased levels in infected macrophages (24 h of infection) compared to uninfected macrophages (Figure 3).

Melatonin treatment (30 nM) reduced the levels of IL-6 (2-fold), MCP-1/CCL2 (5-fold), and RANTES/CCL5 (6-fold) compared to vehicle treatment after both 4 and 24 h of infection, while both vehicle and melatonin treatment reduced the levels of IL-10, MIP-2/CXCL2, and KC/CXCL1 (Figure 3). Our data demonstrated that *L. amazonensis*-infection and melatonin could regulate the synthesis of these cytokines and chemokines.

### Melatonin Antagonizes Changes in the miRNA Profile in *Leishmania amazonensis*-Infected Macrophages

**Figure 4:**
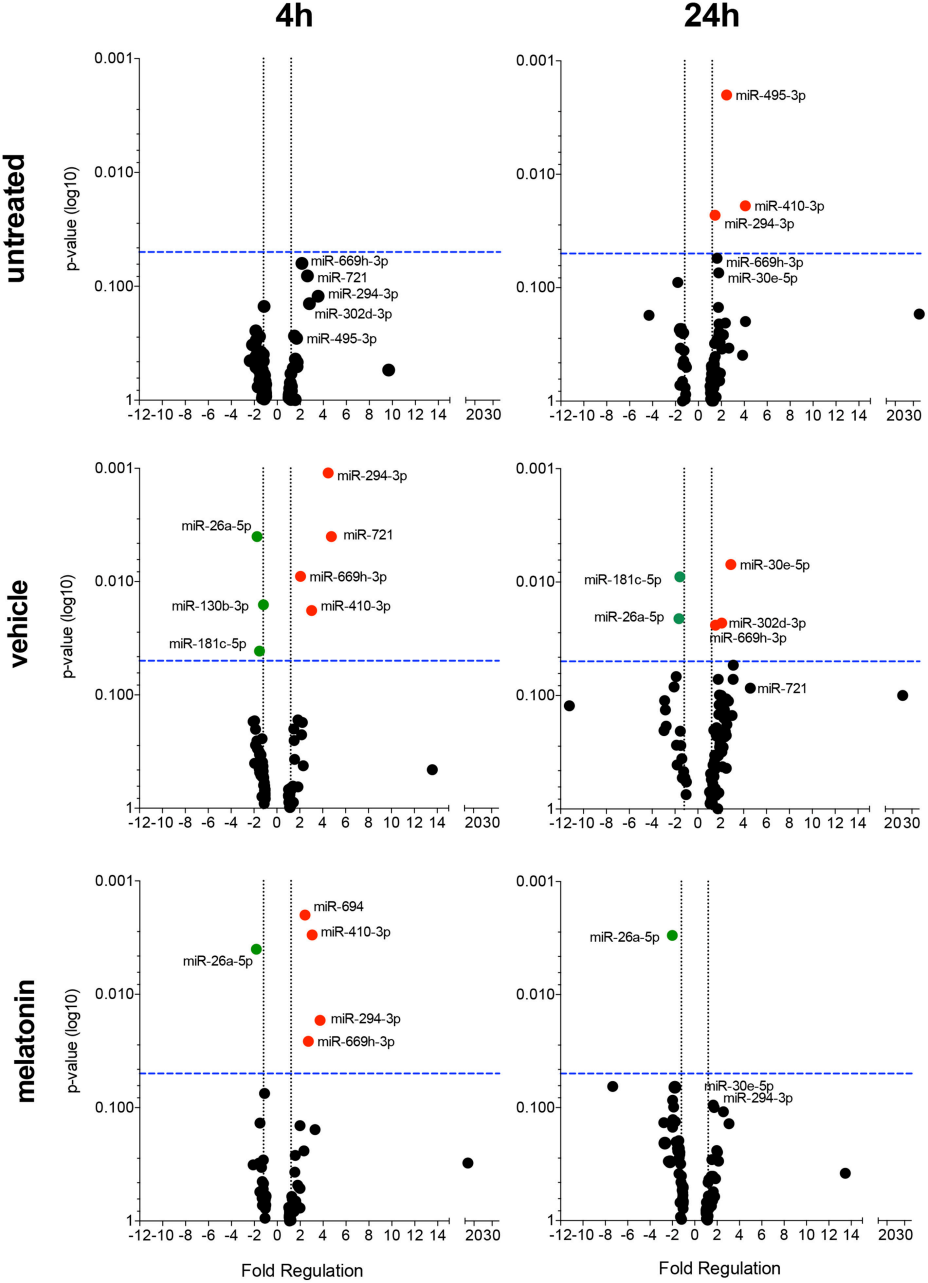
Volcano plot showing melatonin modulation of miRNA profile in macrophages infected with *L. amazonensis* in a time dependent manner. Each dot represents one miRNA in untreated, vehicle or melatonin (30 nM) treated in macrophages infected for 4 or 24 h with *L. amazonensis*. The red dots indicate up-regulated miRNA and green dots indicate down-regulated miRNAs. Blue dotted line corresponds to *p* = 0.05, log 10. The relative up- and down-regulation of miRNAs, expressed as boundaries of 1.5 or −1.5 of Fold Regulation, respectively. *p*-value was determined based on unpaired two-tailed Student's *t*-test. The data are representative of three independent experiments (*n* = 3–4).

The effect of melatonin on the miRNA expression profile was also evaluated after 4 and 24 h of infection. In untreated and 24 h-infected macrophages, only miR-294-3p, miR-410-3p, and miR-495-3p appeared upregulated. In vehicle-treated macrophages at 4 h of infection, miR-294-3p, miR-410-3p, miR-669h-3p, and miR-721 appeared upregulated, while miR-26a-5p, miR-130b-3p, and miR-181c-5p appeared downregulated (Figure 4, Supplementary Table 1). In addition, at 24 h of infection, the expression of miR-302d-3p, miR-30e-5p, and miR-669h-3p appeared upregulated, while miR-181c-5p and miR-26a-5p appeared downregulated.

Melatonin treatment and 4 h of *L. amazonensis* infection induced the expression of miR-294-3p, miR-410-3p, miR-694, and miR-669h-3p, while miR-26a-5p expression appeared reduced (Figure 4). Additionally, melatonin treatment at both 4 and 24 h of *L. amazonensis* infection reduced the expression of miR-26a-5p (Figure 4). However, only miR-694 was exclusively affected by melatonin treatment since it did not appear in vehicle treatment at early infection (Figure 4). These data indicated that melatonin blocked the upregulation of miR-721 and the downregulation of miR-130b-3p and miR-181c-5p after vehicle treatment at 4 h of infection, as well as the upregulation of miR-30e-5p, miR-302d-3p, and miR-669h-3p at 24 h of infection (Figure 4).

Our data demonstrated that melatonin treatment could modulate the miRNA profile of *L. amazonensis*- infected macrophages.

### Inhibition of miR-181c-5p, miR-294-3p, miR-30e-5p, and miR-302d-3p Reduces the Infectivity of *Leishmania amazonensis* by Modulation of *Nos2, Tnf, Mcp-1/Ccl2*, and *Rantes/Ccl5* mRNA Expression

Since miR-181c-5p, miR-294-3p, miR-30e-5p, and miR-302d-3p appeared modulated in both vehicle and melatonin treatment, we performed validation assays to determine the impact of those miRNAs modulation on mRNA expression during infection.

Firstly, we performed *in silico* analysis, and based on a miRecord search, we identified miR-181c-5p, miR-294-3p, miR-30e-5p, and miR-302d-3p targeting thousands of mRNAs (Supplementary Tables 2–5). Among these interactions and trial to focus on L-arginine metabolism and NO, cytokine and chemokine production, we identified that these miRNAs could target the following mRNAs: *Nos*2, *Tnf*, *Mcp*-1/*Ccl2*, and *Rantes/Ccl5*. Then, we performed *in vitro* validation through miRNA inhibition of infection-induced expression at 4 and 24 h. The miR-495-3p, miR-694, and miR-721 did not present *in silico* prediction on these mRNA, with exception of miR-721/*Nos*2 validated target (Muxel et al., 2017b), then we did not perform the validation assay (Supplementary Tables 6–8).

**Figure 5:**
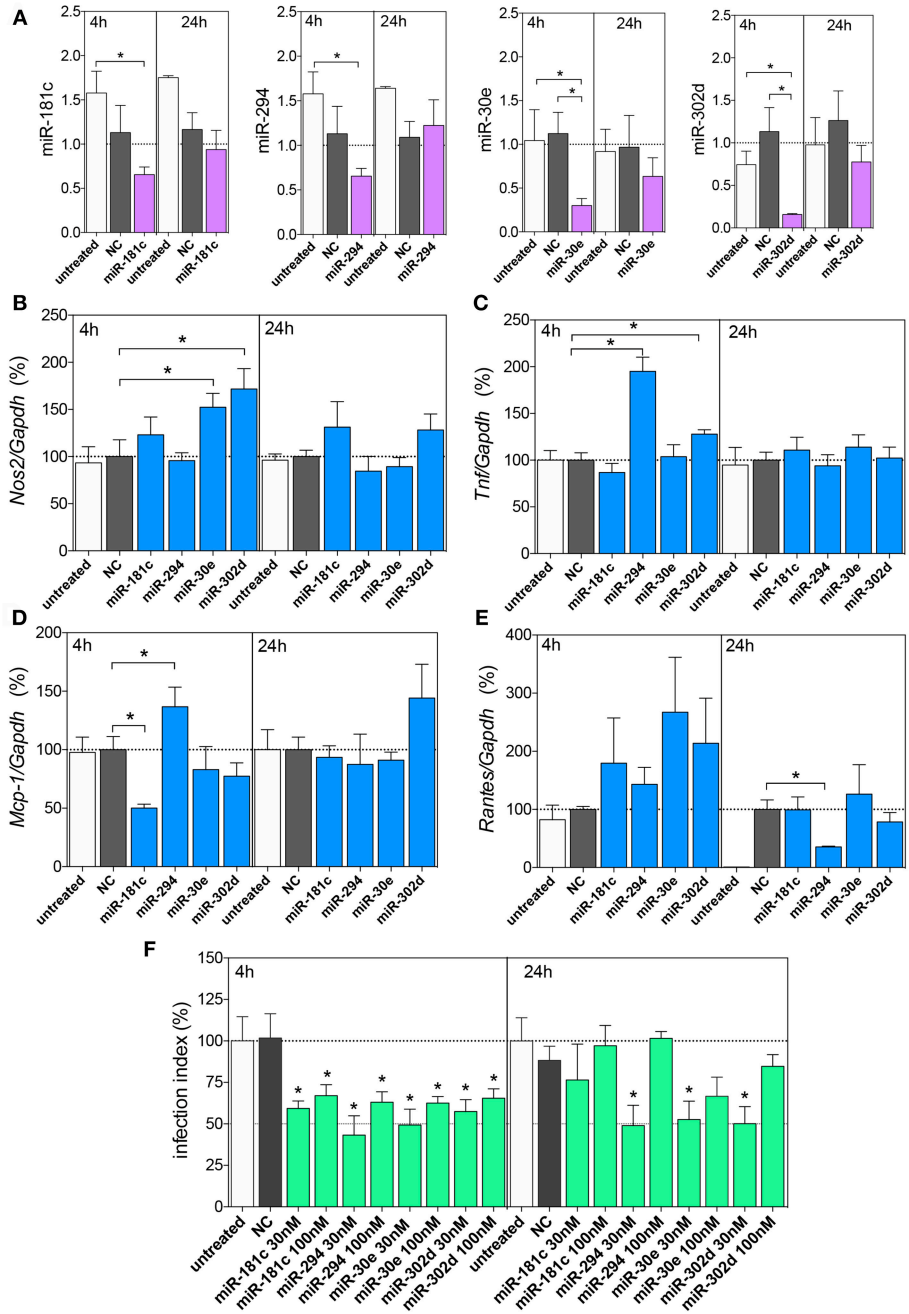
Functional analysis of miR-181c, miR-294-5p, miR-30e, and miR302d inhibition. Macrophages (5 × 10^5^) were transiently transfected with the negative control (NC, gray bars), 30 or 100 nM of miR-181c-5p, miR-294-3p, miR-30e-5p, or miR-302d-3p inhibitors (purple bars), or left non-transfected (untreated, white bars). After 36 h of incubation, the cells were infected with *L. amazonensis* (MOI 5:1) for 4 and 24 h. The inhibition with 100 nM of NC or miRNAs were performed to evaluated miR-181c-5p, miR-294-3p, miR-30e-5p, or miR-302d-3p expression **(A)** and for *Nos2* **(B)**, *Tnf*, **(C)** *Mcp-1* **(D)**, and *Rantes* **(E)** mRNA expression, by qPCR; the inhibition with 30 or 100 nM of NC or miRNAs were also performed for infectivity evaluation **(F)** by microscopy analysis, counting the numbers of infected macrophages and amastigotes per macrophage (*n* = 1,000 macrophages/treatment). The values of miRNAs were represented by Fold change using SNORD95, as a normalizing endogenous control. The values of mRNAs were normalized (100%) based on the average values of the negative control (NC) at 4 h of infection. Each bar represents the average ± SEM of the values obtained in three independent experiments (*n* = 4–6). Statistical significance was determined based on unpaired two-tailed Student's *t*-test. In infectivity analysis **(F)**, ^*^p < 0.05, compared to negative control (NC) infected macrophages.

According to miRNA inhibition assays, we observed reduced levels of these miRNAs at 4 h, but not at 24 h of infection compared to untreated or negative control -transfected macrophages, indicating the efficiency of miRNA inhibition (Figure 5A).

Further, we evaluated the miRNA inhibitions and *Nos*2 mRNA expression levels, and we observed increased levels after inhibition of miR-30e-5p and miR-302d-3p compared to the negative control group (Figure 5B), indicating the interaction of these miRNAs with *Nos2*. The *Tnf* mRNA expression showed increased levels after inhibition of miR-294-3p and miR-302d-3p compared to those in the negative control group (Figures 5C,D), indicating the interaction of these miRNAs with *Tnf*. The *Mcp-1* mRNA expression showed increased levels after inhibition of miR-294-3p (Figure 5D), indicating the interaction of this miRNA with *Mcp-1*. Interestingly, *Mcp-1* mRNA expression showed decreased levels after inhibition of miR-181c-5p (Figure 5D).

Since we observed no statistically significance in miRNA inhibitions at 24 h of infection, we did not infer a role for an interaction between miR-294-3p and *Rantes/Ccl5* mRNA targeting (Figure 5E).

The impact of miRNA inhibition assays were also evaluated in infection index and according to the Figure 5F, we observed that the inhibition of miR-181c-5p, miR-294-3p, miR-30e-5p, and miR-302d-3p at 30 or 100 nM inhibitors reduced the infection index at 4 h of infection, but only 30 nM of miR-294-3p, miR-30e-5p, and miR-302d-3p inhibitors presented reduction in the infection index at 24 h of infection (Figure 5F).

Altogether, the present data indicated that modulation of macrophage infection by melatonin is dependent of these miRNA expressions and therefore on changes in the cell defense program, rather than on only isolated effects at one enzyme or receptor.

## Discussion

Macrophages are essential components of innate immunity and are capable of differentiating into cells with a wide range of functions. These cells are able to respond to different stimuli, such as microbial molecules, damaged cell components, co-stimulatory molecules, cytokines, and chemokines by changing their phenotypes (Mosser and Edwards, 2008; Zhang and Mosser, 2008). In addition, melatonin can act as a pro- or anti-inflammatory agent depending on cell activation state and/or cell type. Melatonin also primes macrophages to a phenotype that reduces *L. amazonensis* infectivity (Laranjeira-Silva et al., 2015).

Melatonin can also act in the modulation of gene expression through transcriptional and posttranscriptional mechanisms, and these intricate regulatory mechanisms interfere with melatonin production. Several studies have shown the role of melatonin in inhibiting arginine uptake via CAT2B (Laranjeira-Silva et al., 2015), CAT1 (Gilad et al., 1998; Deng et al., 2006; Nair et al., 2011), and NOS2 activity (Xia et al., 2012). In contrast, our work showed that melatonin treatment of BALB/c BMDMs increased *Nos2* mRNA expression and NO production during infection, leading to a decreased infection index. Moreover, *Nos2* expression and NO production kill parasites and result in resistance to infection (Ghalib et al., 1995; Wei et al., 1995; Vieira et al., 1996; Wilhelm et al., 2001; Yang et al., 2007; Ben-Othman et al., 2009; Srivastava et al., 2012; Muxel et al., 2017b, 2018a,b).

In this work, we showed that melatonin treatment reduced IL-6, RANTES, MCP-1 and MIP-2 protein levels in infected macrophages, which could correlate with macrophage activation and cell recruitment to the inflammation site. In contrast, melatonin reduced IL-10 levels, which could correlate with immune response modulation induced by infection and pathogenesis. The melatonin treatment was previous described in the μM-mM range concentrations inhibiting IL-1β, TNF-α, IL-6, IL-8, IL-13, and IL-10 and attenuating NOS2 activation induced by LPS (Zhou et al., 2010; Xia et al., 2012). Additionally, melatonin treatment increased TNF-α, IFN-γ, and IL-12 production but reduced NO levels during *Trypanosoma cruzi* infection (Santello et al., 2008a,b). A recent study demonstrated that melatonin also inhibits the production of proinflammatory cytokines, such as TNF-α, IL-6, and IL-12, by TLR-9-stimulated peritoneal macrophages through ERK1/2 and AKT pathways (Xu et al., 2018). However, melatonin in the pM-nM range promotes increased phagocytosis of fungus-derived particles (zymosan) by macrophages (Muxel et al., 2012) and can also reduces the entrance and replication of *L. amazonensis* in peritoneal macrophages (Laranjeira-Silva et al., 2015).

Here, we show that *L. amazonensis* infection of BALB/c untreated-macrophages promoted a reduction in IL-1α production and did not alter IL-1β, IL-6, TNF-α, or IL-12 protein levels. However, in C57BL/6-macrophages, the levels of *Il1b, Tnf*, *Il10*, and *Il6* receptor transcripts increase during infection (Muxel et al., 2018b). IL-1β plays a role in macrophage activation, increasing NO production and leading to host resistance to *Leishmania* infection (Lima-Junior et al., 2013). Also, IL-1 receptor signaling induces NF-kB activation (Ikeda and Dikic, 2008; David et al., 2010; Roh et al., 2014; Fletcher et al., 2015), suggesting a negative regulation of inflammatory cytokine production in infected macrophages. This is in contrast to the upregulation of proinflammatory cytokine gene expression in human macrophages after infection with *L. amazonensis* and *L. major*, such as that of *Il-1b, Tnf*, and *Il-6* (Fernandes et al., 2016), and in murine macrophages after *L. major* infection, in which *Tnf*, *Il-1*, and *Il-6* are upregulated (Dillon et al., 2015). However, melatonin treatment reduced IL-6 levels in infected macrophages compared to untreated infected macrophages. Indeed, melatonin inhibits IL-1β, IL-6, and TNF production mediated via LPS-TLR4 signaling (Xia et al., 2012).

In this work, we showed that another effect of melatonin is related to modulation of miRNA profile imposed by *L. amazonensis* infection. Both miR-294-3p and miR-721 appeared upregulated in vehicle-treated macrophages after 4 h of infection, whereas miR-721 was downregulated in melatonin-treated infected macrophages. Our previous studies showed that these miRNAs reduce *Nos2* expression and NO production, enabling the establishment of infection (Muxel et al., 2017b). Additionally, melatonin blocked the downregulation of miR-130b-3p and miR-181c-5p compared to vehicle. Functional inhibition of miR-181c-5p, miR-30e-5p, and miR-302d-3p increased *Nos2* mRNA expression, impairing infectiveness.

Based on *in silico* analysis of miRNA-mRNA interactions, we performed functional validation through miRNA inhibition. In this way, the inhibition miR-30e-5p and miR-302d-3p increased the levels of *Nos*2 and also the inhibition of miR-294-3p and miR-302d-3p increased *Tnf* levels, which reduced infectivity. Indeed, miR-294 belongs to the miR-291/294 family and controls the cell cycle during embryogenesis (Houbaviy et al., 2003; Wang et al., 2008; Zheng et al., 2011). Previous functional validation of miR-294-3p in *L. amazonensis* infection of BALB/c-macrophages demonstrated that this miRNA targets *Nos*2 mRNA reducing NOS2 expression and NO production impacting in infectivity (Muxel et al., 2017b). Also, miR-294 shares the same putative binding site of miR-302d in *Nos*2 3′UTR, suggesting the competition to interact in the *Nos*2 3′UTR. The signaling via TLR4 by LPS injection on mice downregulates miR-294 levels in blood and lung samples (Fernandes et al., 2016), whereas the miR-294-3p is overexpressed in C57BL/6-macrophages infected with *L. amazonensis* independently of TLR2, TLR4, and MyD88 (Muxel et al., 2018b), suggesting an alternative induction of this miRNA.

The miR-302d encompasses the miR-302/367 cluster that is highly conserved in vertebrates and plays a role in cell proliferation and differentiation (Xia et al., 2012). miR-302d is expressed at the early time and downregulated in late time of exudate during acute inflammation in murine peritonitis induced via TLR2/zymosan stimuli (Recchiuti et al., 2011). In contrast, miR-302d appears at lower levels in plasma of experimental autoimmune encephalomyelitis mouse model and systemic lupus erythematosus and regulates IFN type I gene expression targeting interferon regulator factor-9 (IRF-9) in murine model (Smith et al., 2017). Interestingly, the IRF9, STAT1, STA3, and NF-κB can bind to promoter region of *Nos*2 gene and induce its transcription during *Listeria monocytogenes* infection (Farlik et al., 2010), suggesting both direct and indirect routes for miR-302d regulation of *Nos*2 expression.

Moreover, miR-30e alters cell proliferation, colony formation and invasiveness in cancer cells, interfering with NF-κB/IκBα negative feedback and apoptosis (Jiang et al., 2012; Hershkovitz-Rokah et al., 2015; Zhuang et al., 2017), suggesting a putative role of NF-κB activation during miR-30e-5p inhibition and increased levels of *Nos*2. miR-30e leads to hyperactivation of NF-κB by targeting IκBα 3′UTR, which induces IFN-β and suppresses dengue virus replication (Zhu et al., 2014). miR-30e is overexpressed in *Mycobacterium tuberculosis*-infected THP-1-macrophages (Wu et al., 2017), and in neutrophils of traumatic injured patients correlating to systemic inflammation (Yang et al., 2013).

Additionally, infection and melatonin treatment reduced the levels of KC/CXCL1 and MIP-2/CXCL2 produced by macrophages. These chemokines recruit neutrophils to sites of *Leishmania* infection (Muller et al., 2001). RANTES/CCL5 levels produced by *L. amazonensis*-infected and non-infected macrophages were reduced in melatonin-treated macrophages compared with untreated macrophages. *Rantes/Ccl5* mRNA levels tended to increase after functional inhibition of miR-30e and miR-302d. The cytokines KC/CXCL1, MIP-2/CXCL2, and RANTES/CCL5 are associated with neutrophil, monocyte and lymphocyte recruitment to the inflammatory focus (Schall et al., 1993; Ohmori and Hamilton, 1994; Hornung et al., 1997, 2001; Lebovic et al., 2001), and nocturnal levels of melatonin reduce neutrophil and monocyte migration to inflammatory sites *in vivo* (Lotufo et al., 2001, 2006; Tamura et al., 2010; Marçola et al., 2013).

Interestingly, MCP-1/CCL2 levels were enhanced after infection with *L. amazonensis*, but melatonin treatment reduced this chemokine production in infected macrophages. Melatonin (pretreatment with 100 μM for 4 h) also reduces MCP-1/CCL2 levels and the levels of RANTES/CCL5 produced by PBMCs stimulated with LPS (Park et al., 2007). Corroborating the role of melatonin in miRNA modulation in infected macrophages, inhibition of miR-294-3p increased *Mcp-1* mRNA levels, impacting *Leishmania* infectivity. The validation of interactions of these miRNAs/mRNAs can be explored in the future.

Our data confirmed the previous idea that *Leishmania* infection may regulate cytokine, chemokine and NO production to prevent or delay macrophage activation, allowing parasite entrance and replication. These data reinforced the importance of studying miRNA expression in *L. amazonensis* infection and its role in macrophage activation. Additionally, we demonstrate that melatonin treatment can modulate the miRNA profile and consequently alter the activation phenotype of infected macrophages.

## Data Availability

All datasets generated for this study are included in the manuscript and/or the supplementary files.

## Ethics Statement

Experimental protocols using animals were approved by the Animal Care and Use Committee of the Institute of Bioscience of the University of São Paulo (CEUA 169/2012 and 233/2014). This study was carried out in strict accordance with the recommendations in the guide and policies for the care and use of laboratory animals of the state of São Paulo (Lei Estadual 11.977 de 25/08/2005) and the Brazilian government (Lei Federal 11.794 de 08/10/2008).

## Author Contributions

JF, JA, StM, RZ, and SaM performed experiments. JF, JA, StM, RZ, RM, LF-W, and SaM analyzed data and statistics. SaM and JF prepared the figures. JA, JF, LF-W, and SaM wrote the manuscript. All authors reviewed the manuscript.

### Conflict of Interest Statement

The authors declare that the research was conducted in the absence of any commercial or financial relationships that could be construed as a potential conflict of interest.

## Supplementary Material

Supplementary Figure 1 *Leishmania* infectivity in melatonin-treated macrophages. BALB/c macrophages (2 × 10^5^ cells) were pre-incubated for 1, 2, or 4 h with medium (untreated, white bar), vehicle (ethanol 0.0005%, gray bar), or 3 (blue bar) or 30 (light blue bar) nM of melatonin. After, macrophages were infected with *L. amazonensis* (MOI 5:1) and analyzed after 4 and 24 h. **(A–C)**—percentage of infected macrophages; **(D–F)**—number of amastigotes per infected macrophage; **(G–I)**—infection index (rate of infected macrophages multiplied by the number of amastigotes per infected macrophage). Each bar represents the mean ± SEM of three independent experiments (*n* = 5–8). ^*^*p* < 0.05, comparing the melatonin treatment with the vehicle-treated macrophage at the same concentration and time.

Supplementary Table 1 miRNAs Up-Down Regulation in melatonin and infected *L. amazonensis* infected macrophages compared to uninfected. The relative up- and down-regulation of miRNAs, expressed as boundaries of 1.5 and −1.5 of Fold Regulation, respectively. *p* < 0.05 was considered statistically significant. *p*-value was determined based on two-tailed Student's *t*-test. The data are representative of three independent experiments. Untreated: only 10% FBS RMPI 1640 medium; Vehicle: ethanol 0.0005% in 10% FBS RMPI 1640 medium; Melatonin 30 nM diluted 10% FBS RMPI 1640 medium.

Supplementary Table 2 Predicted mRNA-targets for miR-30e. The miRNA-mRNA interaction was predicted using miRecord tools (http://c1.accurascience.com/miRecords/) based in the integration of various prediction tools: DIANA-microT, MicroInspector, miRanda, MirTarget2, miTarget, NBmiRTar, PicTar, PITA, RNA22, RNAhybrid, and TargetScan/TargertScanS.

Supplementary Table 3 Predicted mRNA-targets for miR-181c. The miRNA-mRNA interaction was predicted using miRecord tools (http://c1.accurascience.com/miRecords/) based in the integration of various prediction tools: DIANA-microT, MicroInspector, miRanda, MirTarget2, miTarget, NBmiRTar, PicTar, PITA, RNA22, RNAhybrid, and TargetScan/TargertScanS.

Supplementary Table 4 Predicted mRNA-targets for miR-294. The miRNA-mRNA interaction was predicted using miRecord tools (http://c1.accurascience.com/miRecords/) based in the integration of various prediction tools: DIANA-microT, MicroInspector, miRanda, MirTarget2, miTarget, NBmiRTar, PicTar, PITA, RNA22, RNAhybrid, and TargetScan/TargertScanS.

Supplementary Table 5 Predicted mRNA-targets for miR-302d. The miRNA-mRNA interaction was predicted using miRecord tools (http://c1.accurascience.com/miRecords/) based in the integration of various prediction tools: DIANA-microT, MicroInspector, miRanda, MirTarget2, miTarget, NBmiRTar, PicTar, PITA, RNA22, RNAhybrid, and TargetScan/TargertScanS.

Supplementary Table 6 Predicted mRNA-targets for miR-495. The miRNA-mRNA interaction was predicted using miRecord tools (http://c1.accurascience.com/miRecords/) based in the integration of various prediction tools: DIANA-microT, MicroInspector, miRanda, MirTarget2, miTarget, NBmiRTar, PicTar, PITA, RNA22, RNAhybrid, and TargetScan/TargertScanS.

Supplementary Table 7 Predicted mRNA-targets for miR-694. The miRNA-mRNA interaction was predicted using miRecord tools (http://c1.accurascience.com/miRecords/) based in the integration of various prediction tools: DIANA-microT, MicroInspector, miRanda, MirTarget2, miTarget, NBmiRTar, PicTar, PITA, RNA22, RNAhybrid, and TargetScan/TargertScanS.

Supplementary Table 8 Predicted mRNA-targets for miR-721. The miRNA-mRNA interaction was predicted using miRecord tools (http://c1.accurascience.com/miRecords/) based in the integration of various prediction tools: DIANA-microT, MicroInspector, miRanda, MirTarget2, miTarget, NBmiRTar, PicTar, PITA, RNA22, RNAhybrid, and TargetScan/TargertScanS.
