# Supplementary figures and images for: Melatonin and Leishmania amazonensis Infection Altered miR-294, miR-30e, and miR-302d Impacting on Tnf, Mcp-1, and Nos2 Expression
Source: Front Cell Infect Microbiol. 2019 Mar 20;9:60. doi: 10.3389/fcimb.2019.00060 (PMC6435487; doi:10.3389/fcimb.2019.00060)

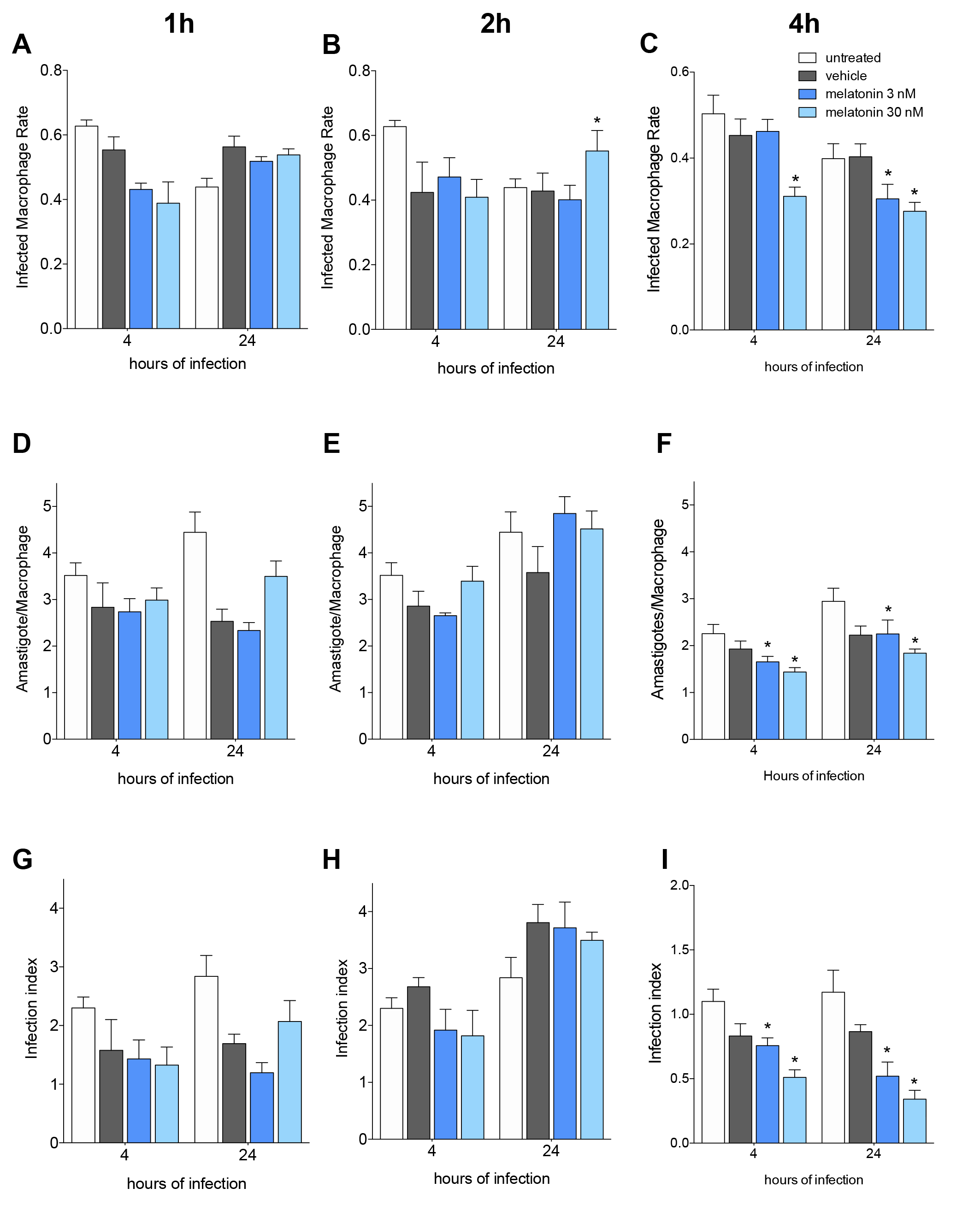

Supplement: Supplementary Figure 1 — Leishmania infectivity in melatonin-treated macrophages. BALB/c macrophages (2 × 10^5^ cells) were pre-incubated for 1, 2, or 4 h with medium (untreated, white bar), vehicle (ethanol 0.0005%, gray bar), or 3 (blue bar) or 30 (light blue bar) nM of melatonin. After, macrophages were infected with L. amazonensis (MOI 5:1) and analyzed after 4 and 24 h. (A–C)—percentage of infected macrophages; (D–F)—number of amastigotes per infected macrophage; (G–I)—infection index (rate of infected macrophages multiplied by the number of amastigotes per infected macrophage). Each bar represents the mean ± SEM of three independent experiments (n = 5–8). *p < 0.05, comparing the melatonin treatment with the vehicle-treated macrophage at the same concentration and time. [file Image_1.TIF]
