# Supplementary material for: Melatonin and Leishmania amazonensis Infection Altered miR-294, miR-30e, and miR-302d Impacting on Tnf, Mcp-1, and Nos2 Expression
Source: Front Cell Infect Microbiol. 2019 Mar 20;9:60. doi: 10.3389/fcimb.2019.00060 (PMC6435487; doi:10.3389/fcimb.2019.00060)
Supplement: Supplementary Table 1 — miRNAs Up-Down Regulation in melatonin and infected L. amazonensis infected macrophages compared to uninfected. The relative up- and down-regulation of miRNAs, expressed as boundaries of 1.5 and −1.5 of Fold Regulation, respectively. p < 0.05 was considered statistically significant. p-value was determined based on two-tailed Student's t-test. The data are representative of three independent experiments. Untreated: only 10% FBS RMPI 1640 medium; Vehicle: ethanol 0.0005% in 10% FBS RMPI 1640 medium; Melatonin 30 nM diluted 10% FBS RMPI 1640 medium. [file Table_1.DOCX]

**Supplementary Table 1: miRNAs Up-Down Regulation in melatonin and infected *L. amazonensis* infected macrophages comparing to uninfected.**

| Hours | **4h** | | | | | | **24 h** | | | | | |
| --- | --- | --- | --- | --- | --- | --- | --- | --- | --- | --- | --- | --- |
| condition | **untreated** | | **vehicle** | | **MEL** | | **untreated** | | **vehicle** | | **MEL** | |
| **ID** | **FR** | **p-value** | **FR** | **p-value** | **FR** | **p-value** | **FR** | **p-value** | **FR** | **p-value** | **FR** | **p-value** |
| let-7a-5p | 1.08 | 0.6970 | 1.87 | 0.2767 | 1.25 | 0.5822 | 4.30 | 0.1754 | 11.24 | 0.1226 | 7.33 | 0.0645 |
| let-7b-5p | 1.23 | 0.8302 | 1.04 | 0.6880 | 1.30 | 0.4486 | 1.67 | 0.2348 | 2.77 | 0.1863 | 2.77 | 0.1359 |
| let-7c-5p | 1.04 | 0.8261 | 1.11 | 0.9776 | 1.09 | 0.6651 | 1.56 | 0.2474 | 2.91 | 0.1110 | 2.04 | 0.1333 |
| let-7d-5p | 1.05 | 0.7280 | 1.07 | 0.6442 | 1.20 | 0.4673 | 1.80 | 0.0900 | 2.08 | 0.0835 | 1.67 | 0.2008 |
| let-7e-5p | 1.21 | 0.4554 | 1.14 | 0.7584 | 1.04 | 0.9384 | 1.57 | 0.3406 | 1.54 | 0.2064 | 1.88 | 0.0985 |
| let-7f-5p | 1.36 | 0.7327 | 1.25 | 0.3915 | 1.07 | 0.8287 | 1.07 | 0.7919 | 1.07 | 0.8866 | 1.42 | 0.2388 |
| let-7g-5p | 1.24 | 0.6023 | 1.51 | 0.3226 | 1.19 | 0.5950 | 1.09 | 0.6456 | 1.25 | 0.4680 | 1.51 | 0.2434 |
| let-7i-5p | 1.44 | 0.6135 | 1.03 | 0.6797 | 1.16 | 0.9137 | 1.34 | 0.2541 | 1.32 | 0.6159 | 1.04 | 0.8128 |
| miR-106a-5p | 1.22 | 0.6502 | 1.16 | 0.8359 | 1.27 | 0.6069 | 1.33 | 0.4866 | 1.45 | 0.3785 | 1.11 | 0.9463 |
| miR-106b-5p | 1.36 | 0.3967 | 1.39 | 0.3383 | 1.06 | 0.8831 | 1.36 | 0.4785 | 1.19 | 0.8197 | 1.02 | 0.8642 |
| miR-1192 | 1.33 | 0.5762 | 1.42 | 0.3868 | 1.14 | 0.9658 | 2.17 | 0.2614 | 2.44 | 0.1093 | 1.08 | 0.8370 |
| miR-126a-5p | 1.56 | 0.9901 | 1.19 | 0.5314 | 1.35 | 0.8260 | 2.66 | 0.3408 | 1.00 | 0.5776 | 1.41 | 0.3845 |
| miR-128-3p | 1.20 | 0.7335 | 1.18 | 0.5200 | 1.03 | 0.7751 | 1.26 | 0.8559 | 1.40 | 0.3609 | 1.49 | 0.2383 |
| miR-130a-3p | 1.14 | 0.7052 | 1.11 | 0.9114 | 1.32 | 0.6291 | 1.26 | 0.6926 | 1.87 | 0.2737 | 1.27 | 0.9256 |
| miR-130b-3p | 1.15 | 0.1501 | 1.17 | 0.0158 | 1.11 | 0.0754 | 1.27 | 0.4403 | 1.89 | 0.0003 | 1.77 | 0.0658 |
| miR-135a-5p | 1.72 | 0.5081 | 2.31 | 0.4215 | 1.99 | 0.5147 | 1.66 | 0.5531 | 2.16 | 0.4246 | 1.78 | 0.6134 |
| miR-140-5p | 1.51 | 0.6651 | 1.55 | 0.4491 | 1.23 | 0.7565 | 1.16 | 0.9994 | 2.32 | 0.1339 | 1.18 | 0.9138 |
| miR-144-3p | 1.04 | 0.8762 | 1.08 | 0.8078 | 1.18 | 0.6578 | 1.13 | 0.4892 | 1.82 | 0.4086 | 2.20 | 0.3013 |
| miR-155-5p | 1.18 | 0.8191 | 1.07 | 0.6348 | 1.46 | 0.8247 | 1.07 | 0.7328 | 1.83 | 0.7188 | 1.04 | 0.7815 |
| miR-15a-5p | 1.85 | 0.5131 | 1.44 | 0.8886 | 1.89 | 0.5044 | 1.11 | 0.8046 | 3.10 | 0.0539 | 2.56 | 0.1086 |
| miR-15b-5p | 1.03 | 0.8980 | 1.23 | 0.6932 | 1.19 | 0.7732 | 1.41 | 0.3106 | 1.19 | 0.5468 | 1.32 | 0.2487 |
| miR-16-5p | 1.11 | 0.6063 | 1.15 | 0.5376 | 1.03 | 0.7379 | 1.12 | 0.4840 | 1.63 | 0.1917 | 1.02 | 0.8019 |
| miR-17-5p | 1.17 | 0.5901 | 1.09 | 0.5967 | 1.28 | 0.7258 | 1.37 | 0.5013 | 1.40 | 0.5165 | 1.11 | 0.9786 |
| miR-181a-5p | 1.23 | 0.7566 | 1.14 | 0.9848 | 1.31 | 0.6399 | 1.39 | 0.4716 | 1.84 | 0.1470 | 1.39 | 0.4228 |
| miR-181b-5p | 1.13 | 0.9719 | 1.16 | 0.8755 | 1.01 | 0.7586 | 1.22 | 0.7499 | 1.13 | 0.5322 | 1.26 | 0.3153 |
| miR-181c-5p | 1.15 | 0.8782 | 1.52 | 0.0410 | 1.21 | 0.2895 | 1.48 | 0.2285 | 1.58 | 0.0092 | 1.23 | 0.4568 |
| miR-181d-5p | 1.58 | 0.4346 | 1.39 | 0.6394 | 1.04 | 0.6489 | 1.64 | 0.2876 | 1.17 | 0.5297 | 1.39 | 0.2789 |
| miR-182-5p | 1.73 | 0.4646 | 1.16 | 0.9820 | 1.79 | 0.4832 | 1.15 | 0.7468 | 1.56 | 0.7617 | 1.31 | 0.6912 |
| miR-186-5p | 1.09 | 0.9088 | 1.02 | 0.7458 | 1.40 | 0.7928 | 1.31 | 0.9981 | 2.41 | 0.2083 | 1.49 | 0.7430 |
| miR-195a-5p | 1.41 | 0.4888 | 1.20 | 0.4707 | 1.06 | 0.8789 | 1.02 | 0.7373 | 1.48 | 0.3359 | 1.02 | 0.6742 |
| miR-19a-3p | 1.82 | 0.2971 | 1.25 | 0.4360 | 1.61 | 0.6681 | 1.11 | 0.8749 | 2.36 | 0.2310 | 2.12 | 0.2984 |
| miR-19b-3p | 1.86 | 0.2455 | 1.62 | 0.3038 | 1.24 | 0.8540 | 1.39 | 0.9970 | 1.90 | 0.2837 | 1.63 | 0.4045 |
| miR-200c-3p | 1.38 | 0.5242 | 1.04 | 0.8879 | 1.26 | 0.5314 | 1.79 | 0.2445 | 1.30 | 0.5964 | 1.07 | 0.5651 |
| miR-20a-5p | 1.05 | 0.9468 | 1.01 | 0.8244 | 1.15 | 0.9735 | 1.41 | 0.5838 | 1.71 | 0.3420 | 1.30 | 0.7622 |
| miR-20b-5p | 1.43 | 0.6638 | 1.11 | 0.8817 | 1.10 | 0.9075 | 1.35 | 0.7647 | 1.79 | 0.3388 | 1.04 | 0.6901 |
| miR-221-3p | 1.18 | 0.705 | 1.11 | 0.8293 | 1.15 | 0.5970 | 1.31 | 0.7272 | 2.96 | 0.2041 | 2.66 | 0.2062 |
| miR-222-3p | 1.14 | 0.9195 | 1.00 | 0.7716 | 1.07 | 0.6609 | 1.21 | 0.7321 | 1.26 | 0.6745 | 1.05 | 0.5972 |
| miR-23a-3p | 1.21 | 0.3968 | 1.30 | 0.2438 | 1.51 | 0.1371 | 1.15 | 0.5756 | 1.89 | 0.0682 | 1.73 | 0.1325 |
| miR-23b-3p | 1.01 | 0.8683 | 1.04 | 0.7242 | 1.06 | 0.9235 | 1.78 | 0.2617 | 1.23 | 0.7977 | 1.10 | 0.4895 |
| miR-26a-5p | 1.13 | 0.9097 | 1.73 | 0.0036 | 1.83 | 0.0038 | 1.28 | 0.2496 | 1.66 | 0.0211 | 2.00 | 0.0026 |
| miR-26b-5p | 2.35 | 0.4514 | 1.87 | 0.2007 | 1.37 | 0.3365 | 1.06 | 0.8481 | 1.07 | 0.8981 | 1.42 | 0.1964 |
| miR-27a-3p | 1.25 | 0.9110 | 1.02 | 0.7742 | 1.36 | 0.6564 | 1.75 | 0.1487 | 2.05 | 0.0995 | 1.47 | 0.4056 |
| miR-27b-3p | 1.24 | 0.9420 | 1.01 | 0.6843 | 1.20 | 0.9868 | 1.61 | 0.2876 | 1.03 | 0.7085 | 1.12 | 0.5377 |
| miR-291a-3p | 1.18 | 0.9873 | 1.84 | 0.6494 | 1.06 | 0.6056 | 1.09 | 0.7475 | 2.49 | 0.2249 | 1.46 | 0.2300 |
| miR-294-3p | 3.55 | 0.1216 | 4.48 | 0.0006 | 3.74 | 0.0166 | 1.46 | 0.0226 | 1.40 | 0.2019 | 1.73 | 0.0998 |
| miR-295-3p | 1.05 | 0.7746 | 1.21 | 0.9510 | 1.09 | 0.8609 | 2.05 | 0.3469 | 2.21 | 0.2847 | 1.07 | 0.7891 |
| miR-29a-3p | 1.31 | 0.5401 | 1.01 | 0.7879 | 1.44 | 0.7734 | 1.23 | 0.9202 | 2.68 | 0.1115 | 1.65 | 0.5546 |
| miR-29b-3p | 2.15 | 0.3266 | 1.21 | 0.4260 | 1.70 | 0.7228 | 1.85 | 0.6626 | 2.99 | 0.1501 | 3.06 | 0.1392 |
| miR-29c-3p | 1.44 | 0.5109 | 1.11 | 0.7907 | 1.52 | 0.7559 | 1.25 | 0.8822 | 3.09 | 0.0721 | 1.86 | 0.4259 |
| miR-301a-3p | 1.46 | 0.4501 | 1.75 | 0.2537 | 1.12 | 0.6401 | 1.62 | 0.7250 | 1.48 | 0.4490 | 1.04 | 0.9629 |
| miR-301b-3p | 1.43 | 0.3769 | 1.29 | 0.4229 | 1.12 | 0.8675 | 1.16 | 0.9638 | 1.51 | 0.6611 | 1.54 | 0.5912 |
| miR-302b-3p | 1.33 | 0.5762 | 1.42 | 0.3868 | 1.14 | 0.9658 | 2.17 | 0.2614 | 2.44 | 0.1093 | 1.08 | 0.8370 |
| miR-302d-3p | 2.79 | 0.1417 | 1.55 | 0.3705 | 1.98 | 0.1435 | 1.15 | 0.5138 | 2.08 | 0.0227 | 1.53 | 0.2893 |
| miR-30a-5p | 1.20 | 0.8887 | 1.27 | 0.5291 | 1.11 | 0.9868 | 1.47 | 0.4037 | 1.65 | 0.2282 | 1.04 | 0.7953 |
| miR-30b-5p | 1.92 | 0.3165 | 1.20 | 0.5310 | 1.01 | 0.7230 | 1.25 | 0.3592 | 1.25 | 0.8827 | 1.15 | 0.9898 |
| miR-30c-5p | 1.39 | 0.7425 | 1.30 | 0.4192 | 1.04 | 0.9030 | 1.24 | 0.6020 | 1.19 | 0.7635 | 1.19 | 0.4027 |
| miR-30d-5p | 1.14 | 0.9757 | 1.16 | 0.6283 | 1.07 | 0.9992 | 1.37 | 0.4328 | 1.86 | 0.1214 | 1.03 | 0.7052 |
| miR-30e-5p | 1.19 | 0.5861 | 1.51 | 0.2533 | 1.58 | 0.2642 | 1.78 | 0.0739 | 2.88 | 0.0071 | 1.65 | 0.0950 |
| miR-322-5p | 1.13 | 0.8299 | 1.08 | 0.6984 | 1.11 | 0.9834 | 1.90 | 0.3027 | 1.95 | 0.2644 | 1.11 | 0.8526 |
| miR-325-3p | 1.87 | 0.4138 | 1.51 | 0.4839 | 1.23 | 0.5244 | 1.54 | 0.9221 | 1.73 | 0.9951 | 1.02 | 0.5039 |
| miR-338-5p | 1.00 | 0.9267 | 1.94 | 0.1690 | 1.60 | 0.3085 | 1.16 | 0.7600 | 1.49 | 0.2755 | 1.98 | 0.0860 |
| miR-340-5p | 1.30 | 0.9600 | 1.07 | 0.7724 | 1.47 | 0.6970 | 1.09 | 0.7622 | 2.53 | 0.1799 | 1.59 | 0.6650 |
| miR-350-3p | 1.37 | 0.6717 | 1.09 | 0.6399 | 1.16 | 0.8779 | 1.49 | 0.7013 | 2.10 | 0.3137 | 1.03 | 0.5118 |
| miR-369-3p | 1.64 | 0.4117 | 1.70 | 0.2984 | 2.10 | 0.3204 | 1.39 | 0.6708 | 1.36 | 0.5317 | 1.04 | 0.7824 |
| miR-384-5p | 1.33 | 0.5762 | 1.42 | 0.3868 | 1.14 | 0.9658 | 2.17 | 0.2614 | 2.44 | 0.1093 | 1.10 | 0.8983 |
| miR-410-3p | 1.49 | 0.2727 | 3.03 | 0.0175 | 3.05 | 0.0032 | 4.09 | 0.0186 | 1.33 | 0.4114 | 1.96 | 0.2396 |
| miR-429-3p | 1.58 | 0.5494 | 1.53 | 0.3363 | 1.19 | 0.6476 | 1.84 | 0.2549 | 1.61 | 0.2200 | 1.08 | 0.6315 |
| miR-466d-3p | 1.00 | 0.7264 | 1.30 | 0.7902 | 1.50 | 0.7787 | 1.92 | 0.5665 | 2.48 | 0.4377 | 1.16 | 0.7283 |
| miR-466k | 1.06 | 0.6839 | 1.39 | 0.5117 | 1.29 | 0.7266 | 1.61 | 0.2291 | 1.23 | 0.8866 | 1.18 | 0.9725 |
| miR-495-3p | 1.68 | 0.2856 | 1.10 | 0.8763 | 1.09 | 0.9760 | 2.46 | 0.0021 | 1.89 | 0.0986 | 1.02 | 0.8288 |
| miR-497-5p | 1.08 | 0.9045 | 2.16 | 0.2250 | 2.33 | 0.2414 | 1.22 | 0.9480 | 2.28 | 0.1579 | 2.03 | 0.2475 |
| miR-568 | 1.67 | 0.7658 | 1.95 | 0.4015 | 1.98 | 0.7674 | 3.85 | 0.3942 | 1.18 | 0.5498 | 2.36 | 0.2992 |
| miR-590-3p | 1.05 | 0.8159 | 1.42 | 0.3868 | 1.14 | 0.9658 | 2.17 | 0.2614 | 2.44 | 0.1093 | 1.08 | 0.8370 |
| miR-669h-3p | 2.15 | 0.0628 | 2.07 | 0.0085 | 2.72 | 0.0259 | 1.63 | 0.0549 | 1.54 | 0.0243 | 1.25 | 0.4334 |
| miR-669k-3p | 1.71 | 0.3710 | 2.08 | 0.1722 | 1.52 | 0.5528 | 1.05 | 0.5026 | 1.03 | 0.7460 | 1.95 | 0.1281 |
| miR-694 | 1.45 | 0.7218 | 2.22 | 0.1749 | 2.42 | 0.0024 | 4.11 | 0.1986 | 2.51 | 0.0001 | 1.19 | 0.4587 |
| miR-712-5p | 1.05 | 0.8696 | 1.83 | 0.1658 | 1.54 | 0.3713 | 1.20 | 0.5154 | 2.84 | 0.1336 | 1.98 | 0.1491 |
| miR-721 | 2.63 | 0.0812 | 4.76 | 0.0042 | 3.30 | 0.1559 | 2.17 | 0.2614 | 4.58 | 0.0859 | 1.27 | 0.7641 |
| miR-743a-3p | 1.55 | 0.2758 | 1.48 | 0.2003 | 1.06 | 0.9454 | 1.80 | 0.2085 | 1.78 | 0.0724 | 1.34 | 0.2610 |
| miR-743b-3p | 1.33 | 0.5762 | 1.33 | 0.4245 | 1.22 | 0.8605 | 2.17 | 0.2614 | 2.44 | 0.1093 | 1.26 | 0.7872 |
| miR-876-3p | 1.33 | 0.5762 | 1.42 | 0.3868 | 1.14 | 0.9658 | 2.17 | 0.2614 | 2.44 | 0.1093 | 1.08 | 0.8370 |
| miR-9-5p | 1.08 | 0.7877 | 1.21 | 0.6644 | 1.05 | 0.7741 | 2.35 | 0.2048 | 1.11 | 0.4891 | 1.21 | 0.6046 |
| miR-93-5p | 1.02 | 0.9531 | 1.04 | 0.7677 | 1.08 | 0.9791 | 1.07 | 0.9602 | 1.31 | 0.4930 | 1.02 | 0.6960 |
| miR-98-5p | 9.69 | 0.5435 | 13.58 | 0.4574 | 16.61 | 0.3075 | 33.24 | 0.1708 | 25.16 | 0.1002 | 13.4 | 0.3810 |

The relative up- and down-regulation of miRNAs, expressed as boundaries of 1.5 and -1.5 of Fold Regulation, respectively. p < 0.05 was considered statistical significant. p-value was determined based on two-tailed Student’s t test. The data are representative of three independent experiments. Untreated: only 10% FBS RMPI 1640 medium; Vehicle: ethanol 0.0005% in 10% FBS RMPI 1640 medium; Melatonin 30nM diluted 10% FBS RMPI 1640 medium.
